# Tuberculosis Trends, Vietnam

**DOI:** 10.3201/eid1305.060904

**Published:** 2007-05

**Authors:** Marleen Vree, Bui Duc Duong, Dinh Ngoc Sy, Nguyen Viet, Martien W. Borgdorff, Frank G.J. Cobelens

**Affiliations:** * KNCV, The Hague, the Netherlands; † Center for Infection and Immunity Amsterdam, Amsterdam, the Netherlands; ‡ National Tuberculosis Program, Vietnam, Hanoi, Vietnam

**Keywords:** Tuberculosis, pulmonary, epidemiology, incidence, Southeast Asia, Vietnam, letter

**To the Editor:** The aims of the global strategy for tuberculosis (TB) control, the directly observed treatment short-course (DOTS) strategy, of the World Health Organization (WHO) are to detect 70% of new smear-positive pulmonary TB cases and cure ≥85% of these detected cases (*1*). If these aims are met in a setting of low prevalence of multidrug-resistant TB and HIV infection, TB incidence is predicted to decrease by >7% annually (*2*). Vietnam has a low prevalence of multidrug-resistant TB (2.3% in 1996–1997 [*3*]) and a low level of HIV infection in the adult population (0.4% in 2003, range 0.2%–0.8% in different settings [*4*]). It is the only country of 22 countries with the highest number of TB cases worldwide that has reached and exceeded WHO targets for TB control since 1997 (*5*,*6*). However, this country has not shown any decrease in TB reporting (*5*).

Reports may not reflect TB incidence if the proportion of cases detected and treated by the National Tuberculosis Program (NTP) varies over time. However, this incidence can be captured by assessing diagnostic efforts. We assessed whether TB case reporting rates in Vietnam are not decreasing because of increased diagnostic efforts in urban, rural, and remote (mountainous) settings. Characteristics of the NTP in Vietnam have been reported (*6*,*7*). The research board of the National Hospital for Tuberculosis and Respiratory Diseases in Hanoi provided scientific and ethical clearance for this study.

Reporting and laboratory register data were collected from 66 randomly selected districts; sampling was stratified to include 20 urban, 30 rural, and 20 remote districts. The NTP defines a suspected TB case-patient as a person with a cough for >3 weeks. A suspected case-patient was a person with a diagnostic sputum smear examination result for acid-fast bacilli by direct microscopy. A total of 20% of suspected case-patients were randomly selected and their data were used. Diagnostic effort was the number of suspect cases per 10,000 persons. A case-patient was a person with new smear-positive pulmonary TB. The reporting rate was the number of cases per 100,000 persons. Population sizes were derived from the national population census of 1999 and projected populations (*8*).

We calculated trends in reporting rates for 1997–2004 by age, sex, and setting (urban, rural, and remote) before and after adjustment for diagnostic effort by using Poisson regression and expressed the average annual percentage change with 95% confidence intervals. Observed trends were adjusted for variation in diagnostic effort over time by standardizing the number of notified cases to the rate of suspected cases in 1997 for that particular setting, age, and sex category.

Total number of cases and suspected cases during 1997–2004 were 28,470 and 138,130 in urban districts, 20,328 and 157,296 in rural districts, and 6,879 and 62,227 in remote districts, respectively. The overall reporting rate per 100,000 persons in 2000 was 78 in urban districts, 64 in rural districts, and 42 in remote districts. The annual change in overall reporting rates was 0.0% in urban districts, 0.4% higher in rural districts, and 0.2% lower in remote districts (Figure). Reporting rates decreased annually in elderly persons (≥55 years of age), and most notably in middle-age women (35–54 years of age, range 5.4%–6.5% in different settings). This was offset by an annual increase among young men (15–34 years of age), notably in urban (2.0%) and mountainous (6.6%) districts. The annual change in overall rates of suspected cases was a 6.2% decrease in urban districts, a 0.5% increase in rural districts, and a 0.1% increase in remote districts. Adjustment for rates of suspected cases did not decrease overall reporting rates and did not fundamentally change our conclusions about the age–sex pattern of the trends. Therefore, increased diagnostic effort did not explain the lack of decreasing reporting rates. The observed trends in reporting rates probably reflect underlying trends in incidence.

**Figure:**
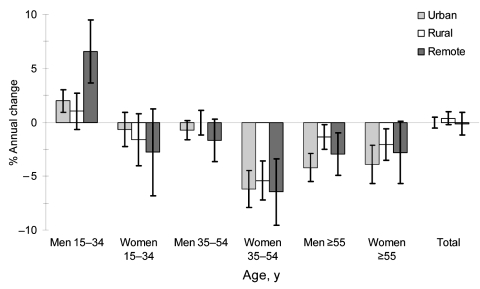
Sex- and age-specific trends in tuberculosis case reporting rates in urban, rural, and remote (mountainous) districts, Vietnam, 1997–2004. Error bars show 95% confidence intervals.

If aims for case detection and cure were met, TB incidence for 1997 through 2004 was predicted to decrease by 44% (*2*). However, no decrease was observed in overall reporting rates in urban, rural, and remote districts in this period in Vietnam. This is explained by an emerging TB epidemic among young adults, particularly in men in urban and remote districts, which causes concern because TB at younger ages tends to reflect recent transmission (*9*).

There are 3 possible explanations for the lack of effect of the DOTS strategy. First, the true case detection rate is lower than estimated. Second, the true cure rate is lower than reported. Third, a mathematical model insufficiently captures the dynamics of TB epidemiology in Vietnam (e.g., because of HIV infection, emergence of the more virulent Beijing TB genotype or risk factors associated with internal migration are underestimated).

Investigation of factors hampering TB control in Vietnam is urgently needed. Efforts are being undertaken to evaluate the effect of HIV on TB trends and to assess case detection in a nationwide TB prevalence survey. The limited effect of the DOTS strategy in Vietnam may be relevant for predicting the effect of this strategy in other countries.
